# The Effect of Technological Conditions on ABE Fermentation and Butanol Production of Rye Straw and the Composition of Volatile Compounds

**DOI:** 10.3390/molecules29143398

**Published:** 2024-07-19

**Authors:** Wojciech Dziemianowicz, Katarzyna Kotarska, Anna Świerczyńska

**Affiliations:** Department of Distillery Technology and Renewable Energy, Prof. Wacław Dąbrowski Institute of Agriculture and Food Biotechnology—State Research Institute, Powstańców Wielkopolskich 17, 85-090 Bydgoszcz, Poland; katarzyna.kotarska@ibprs.pl (K.K.); anna.swierczynska@ibprs.pl (A.Ś.)

**Keywords:** butanol, lignocellulosic biomass, ABE fermentation, enzymatic hydrolysis

## Abstract

The objective of this study was to evaluate the effect of pretreatment and different technological conditions on the course of ABE fermentation of rye straw (RS) and the composition of volatile compounds in the distillates obtained. The highest concentration of ABE and butanol was obtained from the fermentation of pretreated rye straw by alkaline hydrolysis followed by detoxification and enzymatic hydrolysis. After 72 h of fermentation, the maximum butanol concentration, productivity, and yield from RS were 16.11 g/L, 0.224 g/L/h, and 0.402 g/g, respectively. Three different methods to produce butanol were tested: the two-step process (SHF), the simultaneous process (SSF), and simultaneous saccharification with ABE fermentation (consolidation SHF/SSF). The SHF/SSF process observed that ABE concentration (21.28 g/L) was higher than in the SSF (20.03 g/L) and lower compared with the SHF (22.21 g/L). The effect of the detoxification process and various ABE fermentation technologies on the composition of volatile compounds formed during fermentation and distillation were analyzed.

## 1. Introduction

Global energy consumption is expected to increase substantially in the near future, leading to the depletion of fossil fuels, such as coal, natural gas, and oil. Currently, 80% of the energy demand is met by fossil fuels [1]. In addition, an energy crisis, health problems, and climate change caused by excessive carbon dioxide emissions in the environment force the development of more sustainable energy systems based on renewable energy sources. The use and depletion of fossil fuels have necessitated the development of sustainable energy, among which biofuels generated biologically become viable alternatives to oil and other derivate products [2,3]. Among the important biofuels gaining significant prominence in recent years is biobutanol [2,4]. Biobutanol is one of the most promising biofuels due to its environmental benefits and excellent characteristics, such as high energy density and low volatility [5,6]. Further, desirable characteristics of biobutanol are hydrophobicity, low vapor pressure, higher heating value and cetane number, and higher miscibility than ethanol [3].

Butanol can be synthesized via the chemical processing of petrochemical raw materials or by fermentation using anaerobic bacteria, predominantly of the genera *Clostridia* [7]. What is important is that biobutanol produced from the biological route of acetone–butanol–ethanol fermentation has the same chemical properties as n-butanol produced from the petrochemical route. The anaerobic fermentation of sugar-containing materials is the most suitable direction for the production of biobutanol, and *Clostridia* produces an outstandingly high level of biobutanol [8]. This process leads to the production of biobutanol and the formation of ethanol and acetone as the main side products. Hence, this fermentation is also known as ABE fermentation. Different species of *Clostridium* bacteria can produce different amounts of biobutanol through biochemical pathways. They are one of the few organisms able to ferment a large variety of substrates (glucose, sucrose, arabinose, ribose xylose, and starch) to obtain butanol, acetone, and ethanol with a typical ratio of 6:3:1 [9]. However, *Clostridia* are not cellulolytic bacteria and, hence, cannot utilize cellulose as a carbon source. Biobutanol is mainly produced by obligate anaerobe *Clostridium* through sequential metabolic phases, such as acidogenesis, where acetic acid, butyric acid, biohydrogen, and CO_2_ are the main products, and a solventogenesis phase, in which acetone, butanol, and ethanol are produced from the organic acids [9,10].

Among the raw materials currently available for ABE production, lignocellulosic biomass appears as an ideal candidate in terms of the circular economy. Ongoing research has drawn considerable interest in evaluating the potential of numerous lignocellulosic feedstocks [11], which are the most abundant renewable resources on the earth for biofuel production [12]. The selection of feasible substrates plays an important role in butanol production.

Cereal straw may represent an ideal resource for biofuel production, as it is a co-product of food production, and thus, its production does not compete with food generation and will not result in land-use changes [13,14]. Straw as an agricultural residue and is characterized by high potential sugars that can be used in the fermentation process. Despite the many applications for straw, there is a substantial surplus of it that can be used as feedstock in biotechnological production processes. Producing biofuels from agricultural residues will add value to the agricultural industry, increasing the economic attractiveness of green technologies. Utilizing lignocellulosic materials could significantly reduce environmental pollution caused by the accumulation of agricultural wastes [15,16,17], which is why it represents an alternative strategy for biobutanol production [18].

The biochemical route for biobutanol generation relies on the conversion of cellulose (and hemicelluloses) into fermentable sugars using cellulolytic enzymes followed by the fermentation of sugars and transformation of the compounds into butanol. Two main operation strategies can be used: separated hydrolysis and fermentation (SHF) and simultaneous hydrolysis and fermentation (SSF). The first one has its main advantage in the fact that both hydrolysis and fermentation take place under their optimal conditions, whereas a major drawback of the SSF process is a mismatch of optimum temperatures of enzymes and fermenting microorganisms [17,19,20]. Additionally, at high substrate loading, a higher viscosity results in a lower efficiency of heat and mass transfer [21] and hinders enzyme–substrate interaction. High substrate loading also can produce inhibitors, which directly affect butanol yield and productivity [22,23,24]. However, in this simultaneous process (SSF), the number of processing steps and process duration could be reduced, which could lead to a reduction in the overall butanol production cost [25]. Several modifications in the SSF have been suggested, such as the inclusion of a pre-saccharification or prehydrolysis step at the optimum enzyme temperature, which causes effectual liquefaction and reduces the viscosity of the entire slurry [26,27,28]. This method is called consolidation SHF/SSF or PSSF. The main aim of the SHF/SSF is to partially hydrolyze the cellulose to sugars prior to bacterial addition so that butanol production during the initial phase could be increased. In this process, the optimum temperature for enzymatic hydrolysis is applied at the beginning (improving the saccharification) and later, the temperature is set at the optimum range for ABE fermentation [29].

The transformation of fermentable sugars into biofuels requires additional pretreatment to change the lignocellulosic structure and remove lignin [30,31]. Many pretreatment methods such as acidic treatment, alkali treatment, thermal treatment, and physical treatment were investigated [30,32]. Alkaline pretreatment is one of the most effective methods of breaking the ester bonds between lignin, hemicellulose, and cellulose. It refers to the application of alkaline bases, such as sodium hydroxide, NaOH, potassium, KOH, calcium hydroxide, Ca(OH)_2_, and ammonia hydroxide, NH_4_OH, among others [33,34]. Pretreatment can be performed under mild temperature and pressure conditions, minimizing sugar degradation and inhibitory compound formation [35]. The primary outcome of alkaline pretreatment is to break down the ester linkage between the carbohydrate fractions and lignin through which the solubilization of lignin occurs. This, in turn, leads to an increased surface area and increased porosity of the biomass, while decreasing the degree of polymerization and thus enhancing the reactivity of the remaining structural carbohydrates to enzymes [36,37]. Therefore, thermal pretreatment in an alkaline environment was selected for delignification in this research. It should be noted that unlike most yeast strains used in ethanol production, the main advantage of butanol-producing cultures of bacteria is the availability of a wide range of sugar sources, including pentos, hexose, disaccharides, and polysaccharides (starch) [18].

The key objective of this work was to assess the impact of pretreatment and various technological methods of fermentation on the course of the ABE process and the amount of volatile components. ABE fermentation of rye straw (RS) previously treated by thermal pretreatment and enzymatic hydrolysis was conducted using *C. acetobutylicum* DSM 1731. This research assesses the impact of delignification, enzymatic hydrolysis, and detoxification on key fermentation metrics, including rates, yields, and titers. Separated hydrolysis and fermentation (SHF), simultaneous saccharification and fermentation (SSF), and pre-saccharification and simultaneous saccharification with fermentation (SHF/SSF) were investigated at 10% *w*/*v* biomass loading. The course of ABE fermentation under different technological conditions was analyzed and compared to identify the preferable fermentation mode and the effect on the composition of volatile compounds.

## 2. Results and Discussion

### 2.1. Enzymatic Hydrolysis Optimum Conditions and the Composition Analysis of RS

The key step to producing biofuels from cellulosic materials is the hydrolysis process. Due to the crystalline structure and the presence of lignin components in RS, the pretreatment of the raw material was required (i.e., physical, mechanical, and chemical treatments). After pretreatment, the enzymatic hydrolysis of the whole slurry was performed (was not separated into liquid and solid fractions). The enzymatic degradation of cellulose into glucose required the synergistic action of three types of cellulases: endoglucanases, cellobiohydrolases, and β-glucosidases. The enzymes were used together in the mixture of Cellic^®^ CTec2, Viscozyme^®^L, and Pentopan Mono BG. Cellic^®^ CTec2 is characterized by increased β-glucosidase activity. On the other hand, Viscozyme^®^L a multi-enzyme complex (arabanase, cellulase, β-glucanase, hemicellulase), dedicated to a wide range of carbohydrates that guarantee the effective breakdown of polysaccharides. In order increase to the efficiency of enzymatic hydrolysis, a preparation containing an enzyme catalyzing xylan hydrolysis (Pentopan Mono BG) was additionally applied. The conditions for the pretreatment and hydrolysis process were chosen based on the basis of research presented in previous publications [38,39].

The content of cellulose, hemicellulose, and lignin was calculated based on the loss of acid and neutral detergent fibers after alkaline pretreatment and enzymatic hydrolysis. The rye straw was composed of cellulose, 43.45%; hemicellulose, 20.72%; and other (including ash and lignin), 35.83%, as shown in Table 1. Composition analysis (cellulose, hemicelluloses, and lignin) of raw and pretreated rye straw contributed to the partial liquefaction of the lignocellulose complex and the dissolution of the lignin. In this way, the other two polymers (hemicellulose and cellulose) became more accessible for the action of enzymes. Hydrolysis of rye straw with the enzyme mixture resulted in a degradation of polysaccharides to 23.23%. The amount of cellulose, hemicellulose, and lignin after the two-step process of pretreatment (alkaline pretreatment and enzymatic hydrolysis) was 17.95%, 5.28%, and 4.92%, respectively.

The rest was ash and soluble matter or extractives, which include pectins, proteins, and fats, among others. The use of hemicellulase and xylanase for the hydrolysis of the substrate, apart from cellulases, promotes the release of simple sugars and thus improves the economics of the fermentation process. Achieving high concentrations of fermentable sugars is crucial for lignocellulosic butanol. Unfortunately, low sugar concentrations can result in low butanol production. The enzymatic hydrolysis of the substrate generated about 43.12 g/L of monosaccharides, which was suitable for the initiation of biobutanol production in ABE fermentation. The addition of hemicellulase and xylanase to the complex of enzyme preparations resulted in a polysaccharides degradation efficiency of 63.80% (with 58.69% sugar conversion). The sugar concentration depends on diverse lignocellulosic materials and the hydrolysis conditions.

Kumar and Wyman [40] found an increase in the efficiency of glucose and xylose release as a result of the use of hemicellulase. Saha et al. [41] performed the enzymatic hydrolysis of rye straw previously treated with a 0.75% sulfuric acid solution, and the enzyme complex used in this study was cellulase, β-glucosidase, and xylanase. This mixture turned out to be the most advantageous of all those used in the experiment, as evidenced by the obtained conversion of cellulose to glucose of 81% (565 mg of reducing sugar/g of raw material). Gottumukkala et al. [42] reported that a hydrolysate with 39.02 g/L of glucose, 11.35 g/L xylose, and 1.71 g/L arabinose was obtained with dilute acid pretreatment and enzymatic hydrolysis of rice straw. Rice straw had a composition of 47.57% cellulose, 15.75% hemicellulose, and 8.66% lignin. The biomass concentration is an important parameter that influences ABE fermentation [43]. It was observed that with an increasing biomass concentration, the hydrolysates became more viscous and were more difficult to homogenize during treatment, which caused the mass and heat transfer efficiency to decrease. The substrate concentration in the hydrolysate was about 10% *w*/*v*.

### 2.2. Effect of Pretreatment Rye Straw on the Course of ABE Fermentation

**Figure 1:**
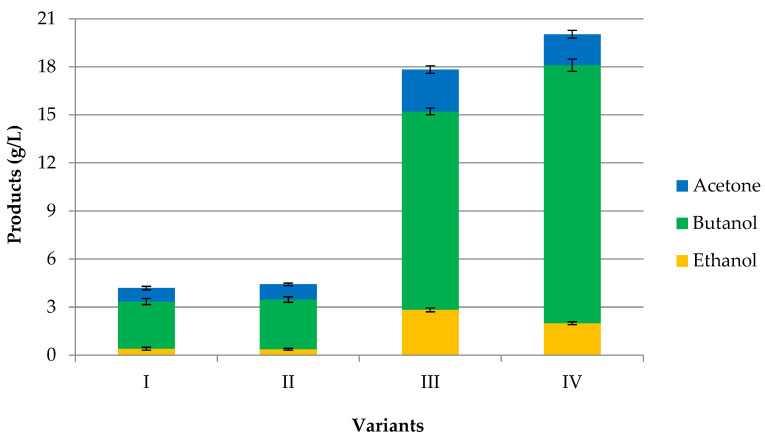
Production of ABE solvents (butanol, ethanol, and acetone) in four different variants: (I) fermentation of pretreated RS by alkaline hydrolysis; (II) fermentation of pretreated RS by enzymatic hydrolysis; (III) fermentation of pretreated RS by alkaline hydrolysis followed by enzymatic hydrolysis; (IV) fermentation of pretreated RS by alkaline hydrolysis followed by detoxification and enzymatic hydrolysis.

In this study, the impact of alkaline hydrolysis (delignification), enzymatic hydrolysis, and detoxification on butanol fermentation were assessed. Four different processes (Variants I–IV) were investigated to produce acetone–butanol–ethanol (ABE) from rye straw (RS) by *C. acetobutylicum* DSM 1731. In order to compare the results obtained in these studies, a batch fermentation (SSF method) was run, in which the concentration of acetone, butanol, and ethanol was measured after 72 h. The processes were the fermentation of pretreated RS by alkaline hydrolysis (Variant I); the fermentation of pretreated RS by enzymatic hydrolysis (Variant II); the fermentation of pretreated RS by alkaline hydrolysis followed by enzymatic hydrolysis (Variant III); and the fermentation of pretreated RS by alkaline hydrolysis followed by detoxification and enzymatic hydrolysis (Variant IV). The number of ABE solvents are compared for Variants I–IV in Figure 1. The four variants were also compared in terms of productivity and yield, as shown in Table 2.

As shown in Figure 1, ABE production was generally improved after all of the three stages of pretreatment. Various concentrations of acetone, butanol, and ethanol were produced, depending on the pretreatment conditions. The pretreatment of rye straw by alkaline hydrolysis followed by enzymatic hydrolysis (Variant III) significantly affected ABE production. In this variant, there was a total solvent production of 17.84 g/L, including 2.62 g/L acetone, 12.38 g/L butanol, and 2.83 g/L ethanol. In addition, this fermentation resulted in an ABE yield of 0.485 g/g, which is a higher value by 64% and 48% compared to Variant I and Variant II.

In the bioconversion of lignocelluloses, two-stage pretreatment (alkaline and enzymatic hydrolysis) has significant importance. Without pretreatment, enzymatic hydrolysis cannot be an appropriate method for the hydrolysis of lignocellulose due to a low yield and rate of conversion. In Variant I, RS was pretreated with a calcium hydroxide solution at 150 °C for 15 min. During 72 h of fermentation, the culture produced 4.18 g/L solvents, i.e., 0.84 g/L acetone, 2.95 g/L butanol, and 0.40 g/L ethanol, resulting in an ABE productivity of 0.058 g/L/h. Similar results were obtained in Variant II. Over the course of 72 h, the culture produced a total solvent concentration of 4.42 g/L, resulting in an ABE productivity of 0.061 g/L/h. The concentrations of acetone, butanol, and ethanol were 0.95, 3.10, and 0.37 g/L, respectively.

One of the limitations of the production of biobutanol is the presence of biomass-derived inhibitory compounds that are generated from the pretreatment process and hydrolysis [44]. These inhibitors such as aliphatic acids, aromatic compounds, and furan derivatives have negative effects on fermentation, affecting the growth and metabolism of the microbes and reducing the efficiency of the strain to convert sugars into solvents. However, phenolics from lignin degradation are much more inhibitory than furan derivatives, and organic acids, as phenolics, can lead to precipitation and irreversible inhibition of enzymes [45,46]. The removal or neutralization of these inhibitors with special physical, chemical, physicochemical, and biological methods can prevent these problems [47,48]. It was reported that using activated carbon in the rice straw hydrolysate, 27% of phenolic compounds were removed [49]. Zhang et al. [50] reported that activated carbon (5.0% *w*/*v*) removed 77.9% of furan derivatives and 98.6% of aromatic monomers. It was observed that AC was effective in removing phenolic acids due to their hydrophobicity. Qureshi et al. [51] investigated the effect of inhibiting compounds, like furfural and HMF, on ABE production from wheat straw hydrolysate using *C. beijerinckii* P260. Studies have shown that the presence of aldehydes was toxic to *Clostridium* bacteria, which led to the inhibition of ABE fermentation and a reduction in the biobutanol content. Also, Cho et al. [6] investigated the effect of phenolic compounds in lignocellulosic hydrolysates on the production of butanol by *C. beijerinckii* NCIM 8052 bacteria. They found that the testing compounds at a concentration of 1 g/L inhibit cell growth by 64–75%, while the production of butanol is completely inhibited.

To increase the fermentability of the hydrolysate in butanol production, the inhibitors were removed with activated charcoal adsorption. It is a simple and economical technique of detoxification, mainly affecting the phenolic compounds. Activated charcoal is characterized by a large surface area, high microporosity, and adsorption capacity, which allows its use as an effective adsorbent for the purification and/or separation of effluents [47]. Table 2 shows the products’ overall yield among others when pretreated RS was detoxified and then subjected to fermentation. Over the course of 72 h (Variant IV), total solvents of 20.03 g/L, including 1.93 g/L acetone, 16.11 g/L butanol, and 1.99 g/L ethanol, were produced. Accordingly, detoxification had a positive effect on ABE fermentation. The activated charcoal adsorption of toxic compounds gave a 12% increase in ABE concentration and a 30% increase in butanol concentration when compared to Variant III without detoxification. It should be noted that Variant IV resulted in the highest productivity and a high yield. After 72 h of fermentation, butanol yield and productivity were 0.402 g/g and 0.224 g/L/h compared to 0.337 g/g and 0.172 g/L/h obtained from the non-detoxified hydrolysate, as shown in Table 2. It has been found that the use of the detoxification process allowed for a better use of fermentable sugars by bacteria, which was confirmed by the different amounts of reducing sugars after ABE fermentation. In Variant IV, the amount of fermentable sugars was 36.56 g/L (84% of fermentable sugars were utilized) after fermentation for 72 h. In the non-detoxified hydrolysate (Variant III), the consumed of fermentable sugars by bacteria for butanol production was 33.28 g/L (78% of fermentable sugars were utilized). These achieved results indicate that detoxification of RS with activated charcoal can enhance the ability of microorganisms to ferment the hydrolysate. The results above showed that activated charcoal adsorption is an effective method to remove the toxic compounds generated during the pretreatment of rye straw.

### 2.3. Effect of the Detoxification Process on the Composition of Volatile Compounds

It is well known that ABE fermentation is biological, and in addition to the mixture of ABE solvents, volatile by-products appear in the fermentation medium. It should be noted that the concentration of these products might vary from pretreatment and fermentation conditions. A reduction in volatile fermentation products from ABE fermentation broth has a significant impact not only on the efficiency of biobutanol production by microorganisms but also allows for more effective operation methods for solvent recovery. The most popular are distillation, adsorption, liquid–liquid extraction, reverse osmosis, thermopervaporation, and gas stripping. Out of these separation processes, distillation is used widely in industries nowadays to separate the components from the mixture [52]. Distillation is a separation technique in which separation occurs due to the difference in the volatilities of separated components. When a mixture containing substances of various volatilities is brought to boiling, the composition of the vapors released will be different than the content of solvents in the boiling liquid.

After ABE fermentation, a distillation process was performed. A set for distillation was used, which consisted of, i.e., a distillation column, distillation flask, and dephlegmator with an adjustable electric contact thermometer to measure the temperature of vapors. Distillates were analyzed on a gas chromatograph. The composition of volatile compounds depended on the pretreatment method, as well as the detoxification process. Table 3 shows the concentration of volatile compounds in the distillates, obtained from ABE fermentation of the hydrolysates with detoxification (Variant IV) and without detoxification (Variant III). Volatiles were identified and quantified (aldehydes, higher alcohols, esters, and methanol) by the comparison of the obtained surface peak area to those obtained for standards. The 10 volatiles were identified in the distillates and have been classified based on their chemical classes into three classes of products. These results suggest that some volatile compounds found in the distillates are formed during distillation due to chemical reactions induced by high temperatures.

As shown in Table 3, the number of aldehydes obtained in Variant IV (with activated charcoal adsorption) was 69% lower (697.04 mg/L) compared to Variant III, which was conducted without detoxification, i.e., 2227.59 mg/L. Among them, acetaldehyde and propionaldehyde were the most abundant. In the non-detoxified hydrolysate, their concentrations were 134.19 mg/L and 2022.45 mg/L, respectively. In the hydrolysate with detoxification, the amount of acetaldehyde and propionaldehyde was lower by 23% and 73%, respectively. The concentration of furfural, which comes from the thermal degradation of the biomass, was 70.95 mg/L (Variant III), and while active charcoal was used, its concentration was lower by 51% to 34.47 mg/L. Based on the obtained results, it can be concluded that the method of RS pretreatment significantly influenced the furfural content in the obtained distillates. The next largest group of volatile compounds were higher alcohols. In the tested samples, 1-propanol, isobutanol, and 2-methyl-1-butanol were determined in the alcohol group. It was observed that carrying out the detoxification process during the pretreatment of the raw material resulted in a reduction in the higher alcohol by 55% (38.13 mg/L) compared to Variant III (85.10 mg/L). The third group was esters (ethyl acetate and isoamyl acetate). In the hydrolysate without detoxification, the concentration of isoamyl acetate was 9.21 mg/L; meanwhile, in the hydrolysate with detoxification, this compound was not detected. The amount of ethyl acetate was 2.64 mg/L (Variant III without activated charcoal adsorption); meanwhile, in the case of Variant IV (with detoxification), it was 0.89 mg/L. In Variant IV, a lower amount of methanol and pyridine were also noted (351.95 mg/L and 11.17 mg/L) compared to Variant III (486.68 mg/L and 33.09 mg/L). The effect of using the activated charcoal adsorption was the reduction in the content of all volatile compounds identified in the distillates.

### 2.4. Influence of Technological Conditions of Butanol Production on the Course of ABE Fermentation and the Concentration of Volatile Compounds

The influence of the butanol production method on ABE fermentation and the content of volatile compounds were investigated. Three different methods to produce ABE from RS by *C. acetobutylicum* DSM 1731 were tested in this study: separate hydrolysis and ABE fermentation (SHF), simultaneous enzymatic saccharification and ABE fermentation (SSF), and pre-saccharification and simultaneous saccharification with ABE fermentation (SHF/SSF).

**Figure 2:**
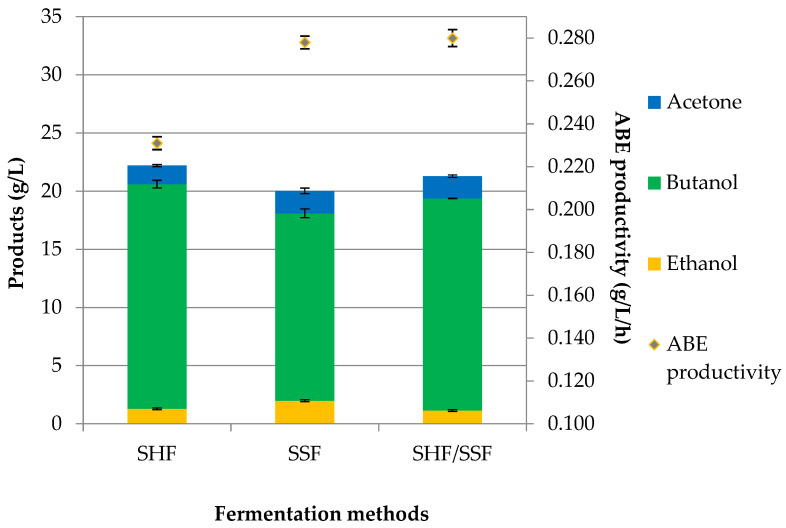
Production of acetone, butanol, and ethanol in the three fermentation methods (SHF, SSF, SHF/SSF) using *C. acetobutylicum* DSM 1731.

The most widely used strategy for lignocellulosic butanol production is the two-step process, where the fermentable sugars were derived from the saccharification of lignocellulosic biomass prior to ABE fermentation. The SHF method ensures the optimum conditions for hydrolysis using hydrolytic enzymes (50 °C, pH 5.0, agitation 140 rpm) and fermentation (37 °C, pH 5.0, no agitation). In the SSF method (simultaneous process), saccharification and ABE fermentation were conducted in a single operation at the same time in a single vessel. The hydrolytic enzymes were added to the reactor at the time of inoculation. This method was performed at 37 °C for 72 h. The third method was consolidation SHF/SSF. The inoculum was introduced into the system after 4 h of pre-saccharification to allow the optimal saccharification process for simple sugar production. The SHF/SSF method reduced the viscosity of the fermentation medium and increased the efficiency of saccharification and fermentation. The ABE production profile from RS in various fermentation methods is shown in Figure 2.

Concerning the SHF method, a total solvent production of 22.21 g/L, including 1.60 g/L acetone, 19.32 g/L butanol, and 1.29 g/L ethanol, was obtained from 40.05 g/L fermentable sugars, resulting in an ABE yield and productivity of 0.554 g/g and 0.231 g/L/h, respectively. The SSF method produced 1.93 g/L of acetone, 1.99 g/L of ethanol, and 16.11 g/L of butanol at 72 h. In comparison to the SHF method, a 10% lower ABE concentration was obtained. In the SSF method, an ABE yield of 0.500 g/g and a productivity of 0.278 g/L/h were calculated. The results are shown in Table 4, which includes reaction time, ABE concentration (g/L), butanol and ABE yield (g/g), and butanol and ABE productivity (g/L/h).

The SHF/SSF method showed that ABE concentration (21.28 g/L) was higher than in the SSF (20.03 g/L) and lower than in the SHF (22.21 g/L). Introducing pre-saccharification in the SHF/SSF increased butanol production from 16.11 g/L (SSF method) to 18.24 g/L, which is equivalent to a 13% increment. In the SHF/SSF with 10% biomass loading, a faster initial (4 h) rate of butanol production was observed than in the SSF due to the monosaccharides needed to initiate butanol production being readily available to the bacteria at the start of fermentation. After 48 h of the process, almost 75% of the total concentration of butanol for the SHF/SSF method and 66% for the SSF method were obtained. The values of acetone, butanol, and ethanol in the SHF/SSF method were 1.91 g/L, 18.24 g/L, and 1.14 g/L, as shown in Figure 2. A maximum butanol concentration was lower than that obtained with the SHF method (19.32 g/L). The highest use of simple sugars was found for the SHF method. In this experiment, the use of fermentable sugars for butanol production was equivalent to 37.21 g/L with, 6.29 g/L sugars remaining after fermentation duration. In the SHF/SSF and SSF methods, 37.05 g/L and 36.56 g/L of fermentable sugars were used, respectively.

**Figure 3:**
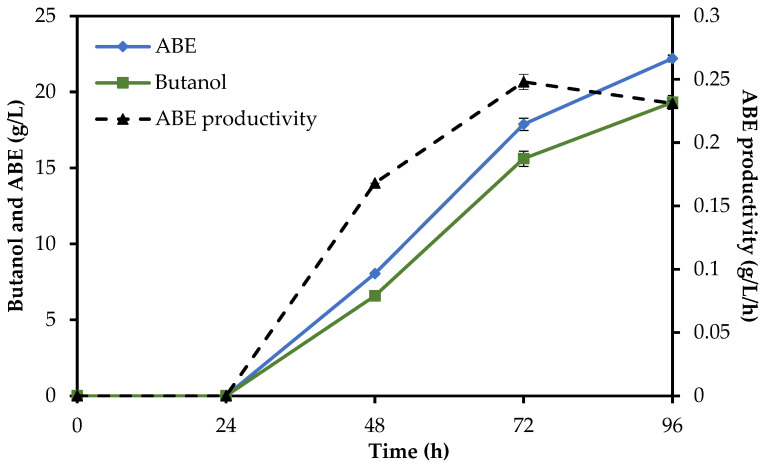
Butanol production, ABE production, and ABE productivity from rye straw under the SHF method.

**Figure 4:**
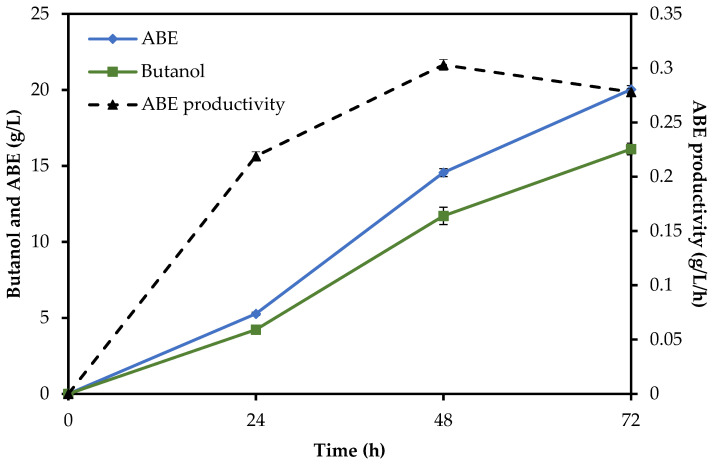
Butanol production, ABE production, and ABE productivity from rye straw under the SSF method.

**Figure 5:**
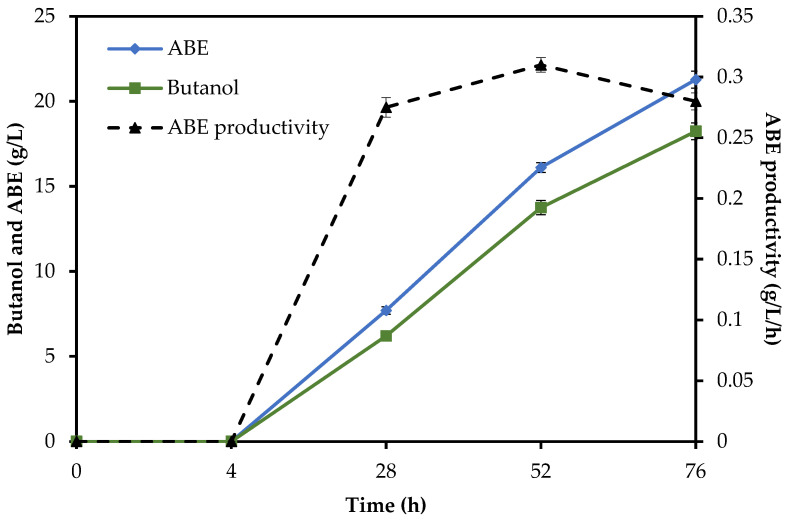
Butanol production, ABE production, and ABE productivity from rye straw under the SHF/SSF method.

To compare the time courses of all methods, the total reaction time of enzymatic hydrolysis plus fermentation was assessed. The SHF method needed a total of 96 h (24 h of enzymatic hydrolysis followed by 72 h of ABE fermentation), while in the SSF and SHF/SSF methods, only 72 h and 76 h were needed, respectively, as shown in Figure 3, Figure 4 and Figure 5. Compared to the SHF method, the time required to complete butanol production for the SHF/SSF method was one day shorter. The lower operation time needed to carry out the valorization process of RS in the SHF/SSF method caused the overall ABE and butanol productivity (0.279 g/L/h and 0.240 g/L/h) to be 21% and 19% higher than the SHF (0.231 g/L/h and 0.201 g/L/h, respectively). ABE yield was 0.531 g/g, while butanol yield was 0.455 g/g, Table 4. ABE yield from the SHF/SSF method was 6% higher when compared to the process SSF (0.500 g/g) and 4% lower when compared to the SHF method (0.554 g/g).

Qi et al. [53] studied the potential of simultaneous enzymatic saccharification and ABE fermentation (SSF) to compare it with separate enzymatic saccharification and ABE fermentation (SHF) using wheat straw as substrate. They found that the SSF performed better than the SHF for ABE production. A high titer of 19.83 (12.64) g/L ABE (butanol) was obtained from 9% (*w*/*v*) wheat straw, with a yield of 173 (110) g/kg raw wheat straw. However, when the biomass loading in the SHF was 10.5%, the ABE (butanol) titer was 17.75 (11.25) g/L, with an ABE (butanol) yield of 133 (84) g/kg raw wheat straw. Also, Valles et al. [45] compared the efficiency of the two different fermentation strategies: the SHF (two-step process) and SSF (one-step process) for biobutanol production from rice straw by *Clostridium beijerinckii* DSM 6422. They reported that the SSF was more efficient than the SHF, with a biobutanol productivity of 0.114 g/L/h. Butanol concentration and productivity at 48 h were, respectively, 8% and 173% higher in the SSF than in the SHF [46].

Based on the number of solvents in the ABE mixture, their volume ratio was calculated. In the SHF method, the ratio of acetone–butanol–ethanol was 0.7:8.7:0.6, with about 87% butanol in ABE composition. Similar results were obtained with the SHF/SSF, where the ratio was 0.9:8.6:0.5, with about 86% butanol. Meanwhile, the A:B:E ratios from the simultaneous saccharification and fermentation method SSF was 1:8:1, with about 80% butanol. These data indicate the fact that the volumetric ratio for all methods shifted towards increased butanol content compared to the “conventional” 3:6:1 [54]. It is important to designate an appropriate volumetric ratio, in which butanol will be the dominant factor in ABE composition. The energy requirement to recover butanol from fermentation broth is influenced by the solvent’s initial concentration in the broth, the expected concentration in the distillate, and the type of recovery systems [55]. Recent developments in ABE fermentation technology have allowed the A:B:E ratios to be precisely controlled. It is possible to designate a specific volumetric ratio such as 6:3:1, 5:14:1, and 0:10:0 to achieve better engine performance, combustion, and emission characteristics [56,57].

In total, 12 volatile compounds were identified and quantitated in the distillates, which were obtained from the SSF, SHF, and SHF/SSF methods. Table 5 shows their concentrations. Carbonyl compounds, including acetaldehyde, propionaldehyde, crotonaldehyde, and furfural, are intermediates in the formation of ethanol and higher alcohols from sugars and amino acids [58]. As shown in Table 5, the number of aldehydes obtained in the SHF, SSF, and SHF/SSF methods were 726.97 mg/L, 697.04 mg/L, and 320.26 mg/L, respectively. Among them, crotonaldehyde was a compound that only appeared in the SHF method (64.15 mg/L). Acetaldehyde and propionaldehyde were present in all methods. Their lowest content was found in the distillate obtained in the SHF/SSF method, which was 84.20 mg/L and 223.22 mg/L, respectively. In the case of the other methods, the concentrations of acetaldehyde were 250.16 mg/L (SHF method) and 103.70 mg/L (SSF method), and the concentrations of propionaldehyde were 405.38 mg/L (SHF) and 538.87 mg/L (SSF). The furfural appeared in the following quantities in the SHF (7.28 mg/L), SSF (34.47 mg/L), and SHF/SSF (12.84 mg/L) methods.

In the tested samples, 1-propanol, isobutanol, and 2-methyl-1-butanol, and 3-methyl-1-butanol were determined in the higher alcohol group. In the SFF and SHF/SSF methods, low contents of these compounds in the distillate were found, which were 38.13 mg/L and 28.61 mg/L, respectively. However, the high content of higher alcohols was noted in the SHF method (1119.13 mg/L). The concentration was higher by 1090.52 mg/L when compared to the SHF/SSF and by 1081 mg/L when compared to the SSF. Among the higher alcohols detected, 2-methyl-1-butanol dominated throughout all fermentations. Depending on the fermentation methods, the concentration of 2-methyl-1-butanol was 830.71 mg/L for the SHF, 24.97 mg/L for the SSF, and 16.66 for the SHF/SSF. 3-methyl-1-butanol was a compound that appeared only in the SHF method (17.07 mg/L).

## 3. Materials and Methods

### 3.1. Substrate

Rye straw was collected from a farm site in Wielki Konopat, Poland. Stalks of cultivated rye plants (with a dry matter content of 94.1%) were used as a substrate in this study. The biomass was dried for 48 h at room temperature, cut into 2 cm long pieces, and grounded by a cutting mill (ZBPP, Bydgoszcz, Poland) to a particle diameter in the range of 0.5–1.0 mm. Afterwards, it was dried in an oven at 80 °C for 48 h until constant weight (until the residual moisture content was less than 7% (*w*/*w*)). The main components in the raw material RS, exhibited by compositional analysis on dry weight (DW), are cellulose (43.45%), hemicellulose (20.72%), lignin (9.41%), and ash with extractive substances (26.42%). Carbohydrate content and lignin in the samples were determined as described by Van Soest [59], in accordance with the ISO 13906 [60] and ISO 16472 [61] methodologies.

### 3.2. Raw Material Preparation

In order to delignify and increase the exposition of cellulose to the hydrolytic enzyme, alkaline pretreatment with a calcium hydroxide solution (prepared by dissolving 0.50 g of Ca(OH)_2_ in 130 mL of distilled water) at 150 °C for 15 min was performed. In every raw material pre-processing, 100 g of rye straw (based on dry weight) was weighed, and 1500 mL of the calcium hydroxide solution and substrate were poured into the high-pressure stainless-steel reactor with a working volume of 2000 mL. The solid substrate concentration was about 10% (*w*/*v*). The start time of the pretreatment process was considered after reaching the desired temperature. In order to decompose the lignocellulosic structure of biomass, a High-Pressure Versoclave Type 3E reactor from Bűchiglasuster was used. Rye straw was subjected to the decomposition process under a high pressure, i.e., up to 4 bar, and a high temperature, i.e., up to 150 °C, for 15 min. Obtaining the set temperature in the reaction vessel was achieved through heaters located in the jacket of the reaction vessel. The controller connected to the reactor was allowed temperature control inside the reactor or heating jacket. An automatic pressure controller and solenoid valves were used, through which the achievement of the conditions of increased pressure in the reaction vessel took place by dosing inert gas (N_2_) into the reactor and the control of the set pressure value. The reactor was additionally equipped with an anchor-type agitator with high torque, i.e., 200 Ncm, designed to work in conditions of up to 250 °C at a maximum pressure of 60 bar. This type of agitator allowed the biomass to be mixed inside the reactor continuously. Upon completion of the processing time, the sample was cooled to 50 °C in a water bath. An unfolded rye straw sample (without separating into the liquid and solid fraction) was neutralized by 1 M H_2_SO_4_ to pH 5.0. The pretreated rye straw was used for the subsequent steps, including saccharification and fermentation.

### 3.3. Enzymatic Saccharification of Pretreated Rye Straw

Three commercial enzymes, (1) Cellic^®^ CTec2, produced from Trichoderma reesei, (2) Viscozyme^®^L, produced from *Aspergillus* sp., and (3) Pentopan Mono BG, produced from Aspergillus oryzae (Novozymes, Bagsværd, Denmark), were used for the saccharification of pretreated rye straw into fermentable sugars. The enzyme activity of Cellic^®^ CTec2 was 150 filter paper FPU/mL, 100 FBGU/g for Viscozyme^®^L, and ≥2500 units/g for Pentopan Mono BG. Saccharification was performed in a shaker incubator (Labwit, Shanghai, China) with an agitation speed of 140 rpm at 50 °C. The pH was adjusted to 5.0 by 3 M NaOH or 1M H_2_SO_4_ before hydrolysis. The yield of polysaccharide degradation is calculated as the sum of the individual steps of the pretreatment.

After the alkaline pretreatment, the adsorption process with activated charcoal and temperature alteration was used to reduce the toxic compounds in the hydrolysate, which presents a high efficiency associated with a low cost. In this method, the optimum temperature, stirring rate, and process time were at 80 ± 2 °C, 150 rpm, and 2 h, respectively. The ratio between activated charcoal mass and hydrolysate volume was 1:5 (20 % active charcoal adsorption).

The sugar conversion was calculated as follows:

(1) $$\alpha_{\mathrm{cvs}}\;=\;[1-\frac{w_{\mathrm{res}}}{w_{\mathrm{all}}}]\;\times \;100\%$$

where α_cvs_ is the sugar conversion, $w_{\mathrm{all}}$ is the cellulose content of the rye straw, and $w_{\mathrm{res}}$ is the residue cellulose content.

### 3.4. Fermentation

#### 3.4.1. Culture Conditions of the Microbial Strain

A *Clostridium acetobutylicum* strain was obtained from the DSMZ culture collection (Braunschweig, Germany). *C. acetobutylicum* DSM 1731 was cultured using the 411 DSMZ medium. The pre-culture medium contained the following substances per liter of distilled water: fresh potatoes (washed, peeled, and sliced), 200.0 g, CaCO_3_, 2.0 g, Na resazurin solution (0.1% *w*/*v*), 0.5 mL, D–Glucose, 6.0 g, and L–Cysteine–HCl × H_2_O, 0.5 g. The medium was purged with nitrogen gas to remove dissolved oxygen and sterilized at 121 °C for 12 min. The initial pH of the medium was adjusted to 7.1 ± 0.1 by 3M NaOH or 1M H_2_SO_4_. This microflora was cultivated in anaerobic vials at 37 °C for 48–72 h, without agitation. For this purpose, a stainless-steel Thermo Scientific™ Oxoid Anaerobic 3.5 L jar was used (Waltham, MA, USA). The creation of anaerobic conditions involved the use of a special sachets Thermo Scientific™ Oxoid AnaeroGen 3.5 L.

#### 3.4.2. Butanol Fermentation

Three methods of ABE fermentations were tested in this study: (1) separate enzymatic saccharification and ABE fermentation (SHF), (2) simultaneous enzymatic saccharification and ABE fermentation (SSF), and (3) consolidation SHF/SSF. All methods used pretreated rye straw as a substrate.

Experiment 1: the SHF was conducted method in a two-step process. Pretreated rye straw was first hydrolyzed into fermentable sugars for 24 h until reaching the maximum hydrolysis efficiency. After hydrolysis at pH 5.0, fermentation was anaerobically initiated at 37 °C in a 2 L stirred bioreactor by 10 % inoculation. No extra nutrients were required.

Experiment 2: SSF experiments were conducted in the simultaneous process, where pretreated rye straw was subjected to conversion into ABE by adding together the enzymes and *C. acetobutylicum* DSM 1731 into the reactor. This method was performed at 37 °C for 72 h, without pH control. No extra nutrients were supplemented.

Experiment 3: a study was conducted using the consolidation SHF/SSF method in order to increase the initial sugar concentration. In the first step (characteristic of the SHF method), the cellulose substrate was subjected to pre-saccharification at the optimum temperature for cellulolytic enzymes (i.e., 50 °C for 4 h). Then, the substrate was cooled and inoculated with *Clostridium* bacteria. The process was conducted at 37 °C for 76 h, where the saccharification and ABE fermentation processes were carried out simultaneously.

The main conditions of the experiment were chosen based on previous research. This study was concerned primarily with the selection of substrate concentration, inoculum size, hydrolysis time, and fermentation duration. The temperature of hydrolysis and fermentation was selected, depending on the enzymes or microorganisms used. The criterion for selecting the conditions for each experiment was to obtain the highest possible content of simple sugars and butanol concentration.

For all methods, the initial pH was 5.0, and the substrate concentration in the hydrolysate was about 10% *w*/*v*. The results from these three processes were compared for the ABE yield and productivity, concentration of solvents, and the composition of volatile compounds in the ABE mixture. The samples were withdrawn every 24 h for ABE concentration and residual sugar analysis. To compare the reaction time of all three types of ABE fermentation, the total reaction time of hydrolysis plus fermentation was assessed.

Fermentation was conducted in a customized 2000 mL bioreactor (1500 mL working volume. Before and after inoculation, the medium was purged with nitrogen gas (N_2_) at a flow rate of 20 mL min^−1^ for 5 min to remove dissolved oxygen and achieve anaerobic conditions. For all samples, the cultivation medium was anaerobically inoculated with 10% (*v*/*v*) *C. acetobutylicum* DSM 1731 stock culture, and the mixture (total volume 1.5 L) was kept at 37 °C. At the bottom of the reactor, there was a valve for sampling the fermentation medium intended for the determination of fermentation parameters. Experiments were conducted in triplicates.

The experimental data were reported as mean ± standard deviation. Tukey’s test was used to evaluate the statistical significance of the differences between groups, considering a confidence level of 95% (*p* < 0.05). The statistical analysis was performed on Statistica 12.0 (StatSoft Inc., Tulsa, OK, USA).

### 3.5. Sample Analysis

The total solid contents and moisture of all samples were determined by drying at 105 °C to a constant weight on the moisture analyzer Radwag MAX 50 (Radwag Balances and Scales, Radom, Poland). The hydrolysis effects were determined based on the concentration of the released reducing sugars using the Lane-Eynon method. The pH was measured using a pH meter (Mettler Toledo, Columbus, OH, USA). Carbohydrate content (cellulose and hemicellulose) and lignin in the samples were determined by the van Soest method [59] using a FOSS Fibertec^®^8000 device (FOSS Analytical A/S, Hillerød, Denmark) equipped with a hot and cold extraction unit. The analysis involved the determination of the content of neutral detergent fiber (NDF), acid detergent fiber (ADF), and acid detergent lignin (ADL). The cellulose content was determined from the difference between the ADF and ADL fractions, and the hemicellulose content was determined from the difference between the NDF and ADF fractions. This was in accordance with the manufacturer’s methodology, such as ISO 13906 [60] and ISO 16472 [61].

After butanol production, the fermentation medium was distilled. Distillate samples were obtained with a glass distillation apparatus equipped with 26 shelves; a distillation flask; a dephlegmator with a water jacket equipped with a contact adjustable thermometer to measure the temperature of vapors; a Liebig condenser; and an electric heating mantle. The concentrations of solvents (butanol, acetone) and volatile compounds were determined using gas chromatography (HP 6890, Shimadzu, Kyoto, Japan) equipped with a CP-WAX 57–CB capillary column (50 m × 250 μm × 0.30 μm, Agilent Technologies, Santa Clara, CA, USA) and a flame-ionized detector. The oven temperature was programmed to increase from 40 °C to 160 °C at a rate of 10 °C/min. The injector and detector temperatures were set at 210 °C and 250 °C, respectively. Pure nitrogen gas was used as a carrier gas with a flow rate of 30.0 mL/min. Volatiles were identified and quantified (aldehydes, higher alcohols, esters, and methanol) by the comparison of the obtained surface peak area to those obtained for standards. The ethanol concentration (g/L) was determined using a Carl–Zeiss refractometer and alcohol tables (Jena, Germany), with an earlier prepared 100 mL sample, using the distillation method. Samples were analyzed in triplicate. ABE productivity (g/L/h) was calculated as the total ABE concentration achieved (g/L) divided by the fermentation time (h). Butanol productivity (g/L/h) was calculated as the maximum butanol concentration achieved in g/L divided by the fermentation time (h). ABE yield was defined by the mass of ABE produced (g) per mass of total simple sugars (g). Productivity and yield were calculated in the same manner for all fermentations presented in this study.

## 4. Conclusions

This study has provided insights into the effect of pretreatment and various technological methods of fermentation on the course of the ABE process and the composition of volatile compounds. The highest concentration of ABE and butanol was obtained from the fermentation of RS pretreated by alkaline hydrolysis, followed by detoxification and enzymatic hydrolysis. ABE fermentation of hydrolysates has been shown to require detoxification with activated charcoal to increase the fermentability of raw material (RS) in butanol production.

Three different technological methods to produce butanol were tested: the SHF, SSF, and consolidation SHF/SSF. It was found that the volumetric solvent ratio for all methods shifted towards increased butanol content compared to the standard 3:6:1 ratio (A:B:E). The butanol in the acetone–butanol–ethanol aqueous solution content was up to 80%. Introducing pre-saccharification in the SHF/SSF and SHF methods improved ABE production. In these two methods, butanol concentration was significantly higher than in the SSF method, while the total reaction time of enzymatic hydrolysis and fermentation for the SHF/SSF and SSF methods was one day shorter compared to the SHF method.

Furthermore, the course of ABE fermentation of different technological methods was analyzed and compared to identify the effect on the composition of volatile compounds. It should be noted that more than 10 volatiles were identified in the distillates, and the concentration may vary from the pretreatment and fermentation methods.

## Figures and Tables

**Table 1:** Chemical composition of RS before and after chemical pretreatment and enzymatic hydrolysis.

Component (%)	Before Pretreatment	After Pretreatment and Hydrolysis
Cellulose	43.45 ± 1.84	17.95 ± 1.18
Hemicellulose	20.72 ± 1.22	5.28 ± 0.76
Lignin	9.41 ± 0.59	4.92 ± 0.98
Other (ash + extractive)	26.42 ± 0.81	71.85 ± 3.49
Total polysaccharides	64.17 ± 2.55	23.23 ± 1.35
Total sugars (g/L)	^1^ NA	43.12 ± 1.05
Sugar conversion	^1^ NA	58.69 ± 1.46

Notes: The table shows the mean values and standard deviations. Extractives—non-structural material from biomass (the soluble matter in the residue). ^1^ NA—not available.

**Table 2:** Parameters from butanol and ABE production received from the fermentation of hydrolysates obtained in four different variants.

Parameters		Variants			
		I	II	III	IV
Butanol	Concentration (g/L)	2.95 ^a^ ± 0.19	3.10 ^a^ ± 0.17	12.38 ^b^ ± 0.21	16.11 ^c^ ± 0.38
	Yield (g/g)	0.208 ^a^ ± 0.014	0.230 ^b^ ± 0.025	0.337 ^c^ ± 0.019	0.402 ^d^ ± 0.012
	Productivity (g/L/h)	0.041 ^a^ ± 0.004	0.043 ^a^ ± 0.003	0.172 ^b^ ± 0.003	0.224 ^c^ ± 0.005
ABE	Concentration (g/L)	4.18 ^a^ ± 0.14	4.42 ^b^ ± 0.22	17.84 ^c^ ± 0.32	20.03 ^d^ ± 0.17
	Yield (g/g)	0.295 ^a^ ± 0.031	0.328 ^b^ ± 0.033	0.485 ^c^ ± 0.018	0.500 ^d^ ± 0.010
	Productivity (g/L/h)	0.058 ^a^ ± 0.003	0.061 ^a^ ± 0.002	0.247 ^b^ ± 0.003	0.278 ^c^ ± 0.004
Sugars before fermentation (g/L)		2.56 ^a^ ± 0.03	6.17 ^b^ ± 0.07	42.55 ^c^ ± 0.15	43.12 ^d^ ± 0.13
Sugars after fermentation (g/L)		1.88 ^a^ ± 0.04	4.59 ^b^ ± 0.11	9.27 ^d^ ± 0.08	6.56 ^c^ ± 0.07

Notes: The table shows mean values and standard deviations. Mean values designated by different letters and placed in the same row differ and are statistically significant at *p* < 0.05; *n* = 3.

**Table 3:** The concentration of volatile compounds in the distillates obtained from ABE fermentation of the hydrolysates with detoxification and without detoxification.

Volatile Compounds	Hydrolysate	
	Variant III without Detoxification	Variant IV with Detoxification
Aldehydes (mg/L):	2227.59 ± 11.02	697.04 ± 5.21
Acetaldehyde	134.19 ± 3.51	103.70 ± 1.35
Propionaldehyde	2022.45 ± 8.27	538.87 ± 4.81
Furfural	70.95 ± 0.95	34.47 ± 1.29
Higher alcohols (mg/L):	85.10 ± 1.23	38.13 ± 0.48
1-propanol	18.23 ± 0.17	2.60 ± 0.04
Isobutanol	30.70 ± 0.34	10.56 ± 0.11
2-methyl-1-butanol	36.17 ± 0.46	24.97 ± 0.29
Esters (mg/L):	11.91 ± 0.23	0.89 ± 0.07
Ethyl acetate	2.64 ± 0.15	0.89 ± 0.07
Isoamyl acetate	9.21 ± 0.47	^1^ NA
Pyridine (mg/L)	33.09 ± 1.18	11.17 ± 0.43
Methanol (mg/L)	486.68 ± 3.27	351.95 ± 2.78

Notes: The table shows mean values and standard deviations. ^1^ NA—not available.

**Table 4:** Comparison of various ABE fermentation methods by *C. acetobutylicum* DSM 1731 using rye straw as a substrate.

Parameters	SHF	SSF	SHF/SSF
Reaction time (h)	96	72	76
ABE concentration (g/L)	22.21 ^c^ ± 0.56	20.03 ^a^ ± 0.17	21.28 ^b^ ± 0.28
Butanol yield (g/g)	0.482 ^c^ ± 0.031	0.402 ^a^ ± 0.012	0.455 ^b^ ± 0.032
ABE yield (g/g)	0.554 ^c^ ± 0.022	0.500 ^a^ ± 0.010	0.531 ^b^ ± 0.028
Butanol productivity (g/L/h)	0.201 ^a^ ± 0.003	0.224 ^b^ ± 0.005	0.240 ^c^ ± 0.003
ABE productivity (g/L/h)	0.231 ^a^ ± 0.007	0.278 ^b^ ± 0.004	0.279 ^b^ ± 0.009
Fermentable sugars (g/L)	37.21 ^c^ ± 0.21	36.56 ^a^ ± 0.14	37.05 ^b^ ± 0.17

Notes: The table shows mean values and standard deviations. Mean values designated by different letters and placed in the same row differ and are statistically significant at *p* < 0.05; *n* = 3.

**Table 5:** The concentration of volatile compounds in the distillates obtained from ABE fermentation of the rye straw from the SSF, SHF, and SHF-SSF methods.

Volatile Compounds	SHF	SSF	SHF/SSF
Aldehydes (mg/L):	726.97 ^c^ ± 4.29	697.04 ^b^ ± 5.21	320.26 ^a^ ± 2.83
Acetaldehyde	250.16 ^c^ ± 1.33	103.70 ^b^ ± 1.35	84.20 ^a^ ± 0.57
Propionaldehyde	405.38 ^b^ ± 4.01	538.87 ^c^ ± 4.81	223.22 ^a^ ± 1.34
Crotonaldehyde	64.15 ± 1.17	^1^ NA	^1^ NA
Furfural	7.28 ^a^ ± 0.89	34.47 ^c^ ± 1.29	12.84 ^b^ ± 0.12
Esters (mg/L):	58.83 ^c^ ± 2.03	0.89 ^a^ ± 0.07	6.67 ^b^ ± 0.07
Ethyl acetate	47.62 ^c^ ± 1.24	0.89 ^a^ ± 0.07	1.65 ^b^ ± 0.02
Isoamyl acetate	11.21 ± 0.54	^1^NA	5.02 ± 0.09
Methanol (mg/L)	647.84 ^c^ ± 3.91	351.95 ^b^ ± 2.78	211.31 ^a^ ± 2.87
Pyridine (mg/L)	37.17 ^b^ ± 0.76	11.17 ^a^ ± 0.43	11.26 ^a^ ± 0.51
Higher alcohols (mg/L)	1119.13 ^c^ ± 7.31	38.13 ^b^ ± 0.48	28.61 ^a^ ± 1.21
1-propanol	29.12 ^c^ ± 1.03	2.60 ^a^ ± 0.04	11.95 ^b^ ± 0.43
Isobutanol	242.23 ± 3.12	10.56 ± 0.11	^1^ NA
2-methyl-1-butanol	830.71 ^c^ ± 5.74	24.97 ^b^ ± 0.29	16.66 ^a^ ± 0.28
3-methyl-1-butanol	17.07 ± 0.20	^1^ NA	^1^ NA

Notes: The table shows mean values and standard deviations. ^1^ NA—not available. Mean values designated by different letters and placed in the same row differ and are statistically significant at *p* < 0.05; *n* = 3.

## Data Availability

Data are contained within the article.
